# Supplementary material for: The Effect of Delivery Matrix on Bifidobacterium animalis subsp. lactis HN019 Survival through In Vitro Human Digestion
Source: Nutrients. 2023 Aug 11;15(16):3541. doi: 10.3390/nu15163541 (PMC10459543; doi:10.3390/nu15163541)
Supplement: Supplementary file 1 [file nutrients-15-03541-s001.zip › nutrients-2520996-supplementary.pdf]

Bifidobacterium animalis subsp. *lactis* HN019, B420

```
TTCGATGGTTCGCACAGTGAGTTGTTCACTCCCCTCAATGAAGCTCCGAAGCCGAGACTTCGGAGATTT
CGTCTCCTCCCTGCGCCGCTGATTGACCTGTTTCCCTCAATGAAGCTCCGAAGCCGAGACTTCGGAGAT
ATTTCAGGCGGCATCAGACC
```

158 bp

Bifidobacterium animalis subsp. *lactis* Bl04, Bi07

```
TTCGATGGTTCGCACAGTGAGTTGTTCACTCCCCTCAATGAAGCTCCGAAGCCGAGACTTCGGAGATTT
CGTCTCCTCCCTGCGCCGCTGATTGACCTGTTTCCCTCAATGAAGCTCCGAAGCCGAGACTTCGGAGAT
GTGATCCCACGTGACGAATCATGGGCCGAGGGATTCCCTCAATGAAGCTCCGAAGCCGAGACTTCGGAG
ATGTCTCATCAGCGGCGCCACGGGTATCAATCTCAATCCCTCAATGAAGCTCCGAAGCCGAGACTTCGG
AGATCCATTCCACCGTTTCGGCGTATTCGCACCACCAGTGCCCTCAATGAAGCTCCGAAGCCGAGACTT
CGGAGATATTTCAGGCGGCATCAGACC
```

372 bp

HN019_F
HN019_R
HN019_P

CRISPR-*cas* repeat

Figure S1. *Bifidobacterium animalis* subsp. *lactis* qPCR amplicons, showing CRISPR-cas repeats that were targeted for assay design. The assay described in this manuscript detects both strains HN019 and B420 (158 bp amplicon) with the same efficiency (~90%). Strains Bl-04 and Bi-07 (372 bp amplicon) are not detectable if either HN019 or B420 are present.

Table S1. *Bifidobacterium animalis* subsp. *lactis* HN019 qPCR assay optimization: panel of bacterial strains tested for amplification at 1 ng of total DNA.

Bacteria	Detection (Yes/No)
Bifidobacterium animalis subsp. *lactis* HN019	Yes
Bifidobacterium animalis subsp. *lactis* B420	Yes
Bifidobacterium animalis subsp. *lactis* Bl-04	Yes *
Bifidobacterium animalis subsp. *lactis* Bi-07	Yes *
Bifidobacterium animalis subsp. *lactis* DSM15954	No
Bifidobacterium breve Bb-03	No
Bifidobacterium longum DGS	No
Bifidobacterium infantis Bi-26	No
Butyrivibrio fibrisolvens DSM 3071	No
Clostridium leptum DSM 753	No
Escherichia coli K88	No
Faecalibacterium prausnitzii DSM 107838	No
Lactobacillus acidophilus NCFM	No
Lacticaseibacillus rhamnosus ATCC53103	No

* Assay detects strains Bl-04 and Bi-07 when each strain is solely present, when mixed (1:1) with either HN019 or B420 the signal corresponds to the amount of present HN019 or B420 only. The amplification efficiency difference is enough to discriminate the two groups of *Bifidobacterium lactis* strains.
